# SARS-CoV-2 Antibody Responses to the Ancestral SARS-CoV-2 Strain and Omicron BA.1 and BA.4/BA.5 Variants in Nursing Home Residents After Receipt of Bivalent COVID-19 Vaccine — Ohio and Rhode Island, September–November 2022

**DOI:** 10.15585/mmwr.mm7204a4

**Published:** 2023-01-27

**Authors:** David H. Canaday, Oladayo A. Oyebanji, Elizabeth M. White, Jürgen Bosch, Clare Nugent, Igor Vishnepolskiy, Yasin Abul, Elise M. Didion, Alexandra Paxitzis, Nicholas Sundheimer, Vaishnavi Ragavapuram, Dennis Wilk, Debbie Keresztesy, Yi Cao, Kerri St. Denis, Kevin W. McConeghy, L. Clifford McDonald, John A. Jernigan, Eleftherios Mylonakis, Brigid M. Wilson, Christopher L. King, Alejandro B. Balazs, Stefan Gravenstein

**Affiliations:** ^1^Geriatric Research Education and Clinical Center, Louis Stokes Cleveland Department of Veterans Affairs Medical Center, Cleveland, Ohio; ^2^Division of Infectious Diseases and HIV Medicine, Department of Medicine, Case Western Reserve University School of Medicine, Cleveland, Ohio; ^3^Department of Health Services, Policy, and Practice, Brown University School of Public Health, Providence, Rhode Island; ^4^Center for Global Health and Diseases, Case Western Reserve University, Cleveland, Ohio; ^5^Center of Innovation in Long-Term Services and Supports, Veterans Administration Medical Center, Providence, Rhode Island; ^6^Division of Geriatrics and Palliative Medicine, Alpert Medical School, Brown University, Providence, Rhode Island; ^7^Ragon Institute of Massachusetts General Hospital, Massachusetts Institute of Technology, and Harvard University, Cambridge, Massachusetts; ^8^Division of Healthcare Quality Promotion, National Center for Emerging and Zoonotic Infections, CDC; ^9^Warren Alpert Medical School of Brown University, Providence, Rhode Island; ^10^Infectious Diseases Division, Rhode Island Hospital, Providence, Rhode Island; ^11^Brown University School of Public Health Center for Gerontology and Healthcare Research, Providence, Rhode Island.

Introduction of monovalent COVID-19 mRNA vaccines in late 2020 helped to mitigate disproportionate COVID-19–related morbidity and mortality in U.S. nursing homes (*1*); however, reduced effectiveness of monovalent vaccines during the period of Omicron variant predominance led to recommendations for booster doses with bivalent COVID-19 mRNA vaccines that include an Omicron BA.4/BA.5 spike protein component to broaden immune response and improve vaccine effectiveness against circulating Omicron variants (*2*). Recent studies suggest that bivalent booster doses provide substantial additional protection against SARS-CoV-2 infection and severe COVID-19–associated disease among immunocompetent adults who previously received only monovalent vaccines (*3*).* The immunologic response after receipt of bivalent boosters among nursing home residents, who often mount poor immunologic responses to vaccines, remains unknown. Serial testing of anti-spike protein antibody binding and neutralizing antibody titers in serum collected from 233 long-stay nursing home residents from the time of their primary vaccination series and including any subsequent booster doses, including the bivalent vaccine, was performed. The bivalent COVID-19 mRNA vaccine substantially increased anti-spike and neutralizing antibody titers against Omicron sublineages, including BA.1 and BA.4/BA.5, irrespective of previous SARS-CoV-2 infection or previous receipt of 1 or 2 booster doses. These data, in combination with evidence of low uptake of bivalent booster vaccination among residents and staff members in nursing homes (*4*), support the recommendation that nursing home residents and staff members receive a bivalent COVID-19 booster dose to reduce associated morbidity and mortality (*2*).

The current extended ongoing study (*5*,*6*) follows 233 volunteer residents of 28 community nursing homes and veterans homes across two states. The median volunteer age was 74 years (IQR = 67–85 years), 53% were female, 79% were non-Hispanic or Latino (Hispanic) White, 19% were non-Hispanic Black or African American, and 1% were of Hispanic ethnicity. Participants had received their primary mRNA vaccination series by February 2021 and the first booster dose within 9 months after completing the primary series; 78% of participants received a second monovalent booster dose within 9 months of the first booster dose. All participants received the bivalent booster during September–November 2022 after its emergency use authorization.

Serum testing occurred a median of 17 days (IQR = 12–25 days) after receipt of all booster doses. Intermediate blood draws occurred 3 months after the monovalent booster among the group that received 2 booster doses and 11 months after the monovalent booster dose among those who had received only 1 booster dose. All participants or their legally authorized representatives provided informed consent approved by Western Institutional Review Board — Copernicus Group.^†^

Approximately three quarters of participants (77%) had a previous SARS-CoV-2 infection confirmed by a polymerase chain reaction or antigen test or based on increases in SARS-CoV-2 antibody levels that could not be explained by vaccination.^§^ Using these methods, the analysis excluded persons with SARS-CoV-2 infection between receipt of their last booster dose and the bivalent booster dose to reduce confounding related to discriminating between antibody increases from infection versus vaccination.

Anti-spike binding antibodies were assessed using a bead-multiplex immunoassay using Wuhan, Omicron BA.1 and BA.4/BA.5 strains (*5*); neutralizing activity was also assessed using a pseudovirus neutralization assay^¶^ with spike protein based on the ancestral Wuhan and Omicron BA.1 and BA.4/BA.5 strains (*5*).

Study participants were stratified by the number of booster doses received before the bivalent booster dose. Within these groups, the geometric mean titers of anti-spike and neutralizing antibodies were measured at three timepoints 1) 2 weeks after the last booster dose, 2) at the most recent blood draw before receiving the bivalent booster dose, and 3) 2 weeks after receipt of the bivalent booster dose. Distributions of values were categorized by timepoint, assay, strain, and 1 versus 2 previous booster doses. To compare values over time given repeated measures within the same subject, a mixed-effects model predicting log-transformed titers was estimated for each subgroup with random intercepts for study subjects. Model-estimated means across the three timepoints were tested. A Bonferroni adjustment was imposed across all the tests performed. In addition, response to the bivalent dose was analyzed using ordinary least squares regression on log-transformed titers assessing effects of 1) previous infection, 2) a second monovalent booster dose before receipt of the bivalent booster dose, and 3) the interaction of the two effects. In the absence of a detected interaction, the model was estimated without the interaction to summarize main effects of previous infection and number of previous booster doses. All analyses were performed using R software (version 4.2.2; R Foundation) and used functions from the linear and nonlinear mixed effects package.

Titers of anti-spike antibody against Wuhan, BA.1 and BA.4/BA.5 and neutralizing antibodies against Wuhan and BA.1 had declined considerably before administration of the bivalent booster dose (Table) (Figure 1) (Figure 2). This decline was statistically significant in mixed-effects models (adjusted p-value <0.05), except in BA.5 neutralization for those with only 1 previous monovalent booster dose (p = 0.105). Receipt of a bivalent booster dose produced substantial increases in model-estimated neutralizing and anti-spike antibody titers to the ancestral strain and Omicron variants compared with those at the intermediate timepoint between receipt of the previous and the bivalent boosters (all p-values <0.001), restoring immunity after waning vaccine- or infection-induced immunity. The bivalent booster also substantially elevated neutralizing antibody titers against the Wuhan, BA.1, and BA.4/BA.5 strains to levels above those achieved 2 weeks after receipt of the most recent booster dose among persons who had received 1 or 2 monovalent mRNA booster doses (p-value range = 0.035–<0.001). These results suggest that neutralizing capacity of antibodies against Omicron strains achieved after receipt of the bivalent booster dose was higher than that for previous monovalent vaccines. In contrast, anti-spike titers against BA.1 and Wuhan strains increased among all participants after receipt of the bivalent booster dose but did not exceed those achieved after the previous monovalent booster dose (Table) (Figure 2). The trend (p = 0.062) suggests that the anti-spike BA.5 titer was higher after the bivalent booster than after only one monovalent booster. No interaction effect of previous infection status and number of booster doses in response to the bivalent vaccine was detected. Receipt of 1 or 2 previous booster doses only substantially affected the anti-spike BA.1 response, where higher anti-spike responses were observed among persons who had received 2 monovalent booster doses than among those who had received only 1 dose.

**TABLE:** Neutralization and anti-spike antibody titers in nursing home residents after previous receipt of 1 or 2 monovalent mRNA COVID-19 vaccine booster doses and before and after receipt of a bivalent booster dose — Ohio and Rhode Island, September–November 2022

Assay	Virus strain	No. of MV booster doses received	GMT (95% CI)*			Adjusted p-value^†^		
						After receipt of BV dose		After last MV dose versus before BV dose
			After last MV^§^ booster dose	Before BV booster dose	After BV booster dose	Versus after last MV dose	Versus before BV dose	
Neut	BA.1	1	153 (87–272)	25 (15–43)	1,205 (675–2,149)	<0.001	<0.001	<0.001
Neut	BA.1	2	924 (621–1,373)	204 (109–384)	1,506 (1,000–2,269)	0.022	<0.001	<0.001
Neut	BA.4/5	1	186 (61–567)	31 (20–49)	1,425 (799–2,539)	0.035	<0.001	0.105
Neut	BA.4/5	2	1,055 (589–1,614)	160 (84–307)	1,964 (1,356–2,842)	0.001	<0.001	<0.001
Neut	Wu	1	848 (574–1,253)	78 (51–121)	2,608 (1,700–3,999)	0.011	<0.001	<0.001
Neut	Wu	2	1,333 (931–1,908)	445 (256–771)	2,594 (1,874–3,589)	<0.001	<0.001	<0.001
Spike	BA.1	1	2,090 (983–4,444)	56 (37–85)	780 (578–1,053)	0.034	<0.001	<0.001
Spike	BA.1	2	1,393 (1,118–1,735)	258 (176–379)	887 (747–1,053)	0.021	<0.001	<0.001
Spike	BA.4/5	1	270 (144–506)	45 (32–61)	960 (726–1,269)	0.062	<0.001	<0.001
Spike	BA.4/5	2	1,014 (840–1,239)	235 (158–351)	993 (837–1,179)	1.0	<0.001	<0.001
Spike	Wu	1	3,554 (2,216–5,699)	126 (82–194)	2,445 (1,755–3,407)	1.0	<0.001	<0.001
Spike	Wu	2	3,786 (3,009–4,765)	816 (504–1,320)	2,725 (2,221–3,343)	0.833	<0.001	<0.001

**Abbreviations:** BV = bivalent; GMT = geometric mean titer; MV = monovalent; Neut = neutralization; Wu = Wuhan.

* Values are geometric mean of titer and 95% CI.

^†^ P-values method: predicted log-transformed using linear mixed-effects model, repeated measures within subject grouped using random subject effect. Estimated time contrasts from these models compared and p-values presented. Given three contrasts over 12 models, added a Bonferroni adjustment for the 36 tests present in table.

^§^ Timepoints: testing after receipt of the MV and BV booster dose a median of 17 days. In the group that received 1 monovalent booster dose, testing before bivalent dose occurred 11 months after receipt of the first booster dose and a median of 48 days before receipt of the bivalent booster dose. In the group that received 2 MV booster doses, testing before the BV dose occurred 3 months after receipt of the second booster dose and a median of 49 days before administration of the BV booster.

**FIGURE 1:**
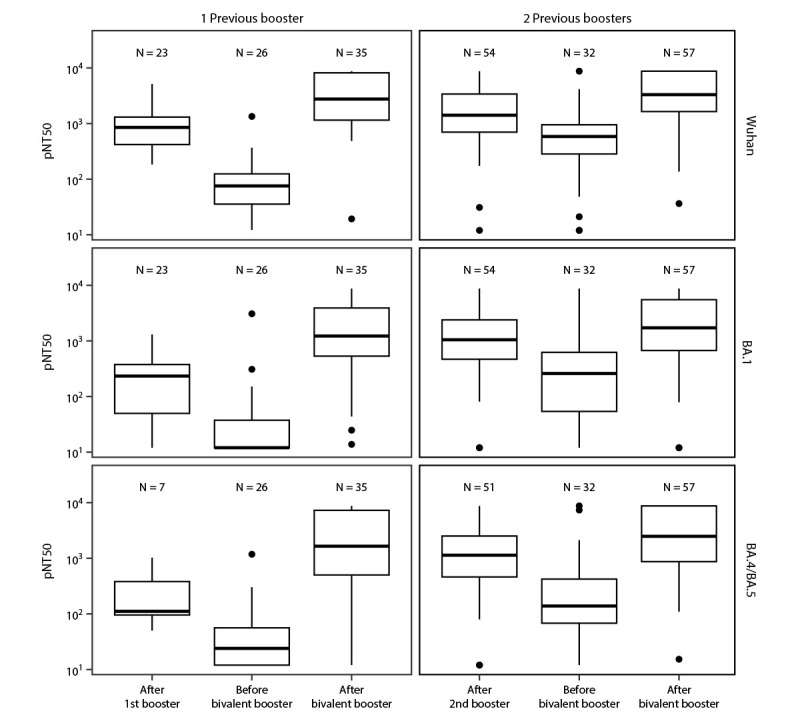
Pseudovirus neutralization assay results for Wuhan (top panels), Omicron BA.1 (middle panels), and Omicron BA.4/BA.5 strains (bottom panels)* in nursing home residents after receipt of 1 (left panels) or 2 (right panels) previous monovalent booster doses and before and after receiving a COVID-19 bivalent booster dose^†^ — Ohio and Rhode Island, September–November 2022^§^ **Abbreviations:** LLD = lower limit of detection; pNT50 = pseudovirus neutralization. * The upper limit of detection of the assay is 1:8,748, and the LLD of the neutralization assay is 1:12. The center line indicates the median, and the bottom and top of the boxes indicate the first and third quartiles, respectively. The lower and upper vertical lines extend from the first and third quartile lines, respectively, to the smallest and largest values no more than 1.5 times the IQR (height of box) away from the first and third quartile values. Values beyond that appear as points. ^†^ Testing after receipt of booster doses occurred a median of 17 days after vaccination in all groups. In the group that received 1 monovalent booster dose, testing before bivalent dose occurred 11 months after receipt of the first booster dose and a median of 48 days before receipt of the bivalent booster dose. In the group that received 2 monovalent booster doses, testing before the bivalent dose occurred 3 months after receipt of the second booster dose and a median of 49 days before administration of the bivalent booster dose. ^§^ Pseudovirus neutralization assay is the method used to measure the ability of antibodies in the serum to neutralize the capability of a virus to enter cells and prevent infection using a pseudovirus containing a nonpathogenic virus core surrounded by a lipid envelope containing the SARS-CoV-2 spike protein surface glycoproteins of the virus strains of interest.

**FIGURE 2:**
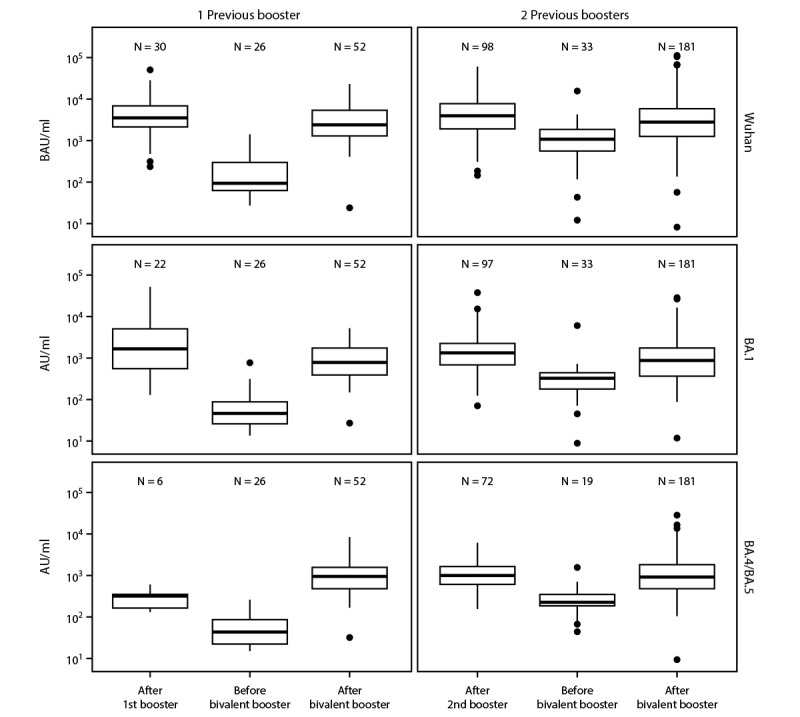
Anti-spike antibody assay results for Wuhan (top panels), Omicron BA.1 (middle panels), and Omicron BA.4/BA.5 strains (bottom panels)* in nursing home residents after receipt of 1 (left panels) or 2 (right panels) previous monovalent booster doses and before and after receiving a COVID-19 bivalent booster dose^†^ — Ohio and Rhode Island, September–November 2022 **Abbreviations:** AU = arbitrary units; BAU = binding antibody units. * Anti-spike levels for BA.1 and BA.4/BA.5 are in arbitrary units (AU/mL) with an internal standard allowing comparison across timepoints in this dataset. Wuhan-anti-spike is in binding antibody units (BAU/mL) that are based on the World Health Organization 20/136 standard. The center line indicates the median, and the bottom and top of the box indicate the first and third quartile, respectively. The lower and upper whiskers extend from the first and third quartile lines, respectively, to the smallest and largest values no more than 1.5 times the IQR (height of box) away from the first and third quartile values. Values beyond that appear as points. ^†^ Testing after receipt of booster doses occurred a median of 17 days after vaccination in all groups. In the group that received 1 monovalent booster dose, testing before bivalent dose occurred 11 months after receipt of the first booster dose and a median of 48 days before receipt of the bivalent booster dose. In the group that received 2 monovalent booster doses, testing before the bivalent dose occurred 3 months after receipt of the second booster dose and a median of 49 days before administration of the bivalent booster dose.

## Discussion

These data show that nursing home residents who received a bivalent COVID-19 mRNA booster vaccine dose mounted substantial antibody titers to the Wuhan and Omicron BA.1 and BA.4/BA.5 variants, irrespective of previous infection or previous receipt of 1 or 2 monovalent booster doses. These findings provide immunologic evidence that the bivalent booster vaccine confers additional protection against SARS-CoV-2 infection among nursing home residents who have previously received only monovalent vaccine.

At the onset of the COVID-19 pandemic, the nursing home population experienced a particularly high case fatality rate (*1*). After national deployment of mRNA vaccines in late 2020, >80% of nursing home residents had completed the primary vaccination series by July 2021, and incidence of COVID-19 and COVID-19–related deaths were subsequently markedly reduced (*7*). After the more recent vaccination recommendations, booster dose coverage has been lower than that initially seen with the primary series, although booster dose coverage among nursing home residents has been higher than that among the general population (*7*). One large study of U.S. adults aged ≥18 years reported that high initial vaccine effectiveness against unplanned care waned across age groups but was more pronounced among immunocompromised persons; no data on vaccine effectiveness among nursing home residents were reported (*8*). A recent study showed that nursing home residents receiving the second COVID-19 monovalent booster dose were protected against SARS-CoV-2 infection, hospitalization, and death during the Omicron period, demonstrating the effectiveness of the monovalent booster among this population during the 60-day follow-up period (*9*). Although serologic studies were not performed in that study, effectiveness of the monovalent COVID-19 booster against the Omicron variant was presumably associated with cross-neutralizing antibody titers generated against both the ancestral Wuhan strain and newer Omicron variants. Similarly, neutralizing antibody titers against the Wuhan, BA.1, and BA.4/BA.5 strains in the present cohort of nursing home residents were higher after the bivalent booster than after the most recent previous monovalent booster, suggesting that the bivalent booster increases and broadens the immune response among nursing home residents.

Data from CDC’s National Healthcare Safety Network show that as of January 8, 2023, one half (50%) of nursing home residents and less than one quarter (22%) of nursing home staff members had received the bivalent booster dose (*4*), highlighting an opportunity to intensify efforts to increase bivalent booster dose coverage among these persons according to current recommendations to reduce the occurrence of severe COVID-19–associated illness, hospitalization, and death. Other studies have demonstrated that antibody levels among nursing home health care workers also markedly increased after booster vaccination (*5*,*10*), reinforcing the recommendation that all eligible nursing home staff members should receive a bivalent booster dose. Furthermore, high staff member vaccination uptake improves outcomes among the residents for whom they care.**

The findings in this report are subject to at least four limitations. First, immunologic findings might not directly translate into real-world reduction in COVID-19 severity. Although binding and neutralizing antibody levels are correlated with protection from SARS-CoV-2 infection at the population level, the absence of precise individual indicators of protection limits interpretability of these data. Second, certain vaccinated participants might have had undetected asymptomatic infection or not have been identified for categorization as having had a previous infection under the laboratory criteria used in this study. This limitation could result in mistakenly attributing the observed immunologic responses to the booster dose rather than the actual recent infection. Third, sample size was relatively limited, with more men included than among the typical nursing home population, primarily resulting from recruitment from two veterans homes with predominantly male populations. However, no substantial difference in immune responses between men and women among the nursing home population has been noted in previous studies (*5*,*6*). Fourth, certain subjects had missing timepoints related to exclusion for recent infection among vaccinated persons, recent enrollment of some participants, unavailability of blood draws at serial timepoints, or incomplete laboratory data. Despite these limitations, this study had adequate power to demonstrate that the bivalent COVID-19 mRNA vaccine booster dose substantially increased anti-spike and neutralizing titers against Omicron sublineages among nursing home residents, supporting current bivalent booster vaccine recommendations.

These findings indicate that nursing home residents can benefit from bivalent booster vaccination, substantially broadening their immune response to tested Omicron variants. Along with nursing home staff members, nursing home residents should stay up to date with recommended COVID-19 vaccines, including receipt of a bivalent booster dose if ≥2 months have elapsed since their last COVID-19 vaccine dose (either a primary series or original monovalent booster) to reduce their risk for infection, severe disease, and death.^††^

**Summary**

**What is already known about this topic?**

Previous COVID-19 monovalent vaccines provided substantial reductions in COVID-19–associated morbidity and mortality among nursing home residents; however, only one half of these residents and one quarter of nursing home staff members have received the COVID-19 bivalent booster dose to date.

**What is added by this report?**

Among nursing home residents in two states, SARS-CoV-2 antibody levels waned within months after vaccination, irrespective of previous SARS-CoV-2 infection, after monovalent booster vaccination. Antibody response broadened after the COVID-19 bivalent booster for vaccinated nursing home residents among those with and without previous infection.

**What are the implications for public health practice?**

All eligible nursing home residents and staff members should follow current recommendations to receive a bivalent COVID-19 booster dose to reduce their risk for SARS-CoV-2 infection, severe COVID-19–associated illness, and death.

## Footnotes

* https://www.cdc.gov/mmwr/volumes/71/wr/mm715152e1.htm?s_cid=mm715152e1; https://www.cdc.gov/mmwr/volumes/71/wr/mm715152e2.htm?s_cid=mm715152e2; https://www.cdc.gov/mmwr/volumes/71/wr/mm7148e1.htm?s_cid=mm7148e1_w

†
https://www.wcgirb.com/

§ Laboratory criteria supporting recent or interval infection were a rise outside of laboratory variance of anti-spike, receptor binding domain, nucleocapsid, and neutralizing assay results not accounted for by vaccination history.

¶ A method used to safely study the effect of antibodies or drugs that neutralize the capability of a virus to enter cells and prevent infection; pseudoviruses contain a nonpathogenic virus core (typically a lentivirus) surrounded by a lipid envelope containing the surface glycoproteins of the virus of interest (i.e., the SARS-CoV-2 spike protein) and the gene for an indicator protein.

** https://www.nejm.org/doi/full/10.1056/NEJMc2115674; https://jamanetwork.com/journals/jamanetworkopen/fullarticle/2799964

††
https://www.cdc.gov/coronavirus/2019-ncov/vaccines/stay-up-to-date.html
